# Blood levels of nicotinic acid negatively correlate with hearing ability in healthy older men

**DOI:** 10.1186/s12877-023-03796-3

**Published:** 2023-02-15

**Authors:** Yoshiko Nakagawa-Nagahama, Masaki Igarashi, Masaomi Miura, Kosuke Kashiwabara, Keisuke Yaku, Yuichiro Fukamizu, Toshiya Sato, Takanobu Sakurai, Takashi Nakagawa, Takashi Kadowaki, Toshimasa Yamauchi

**Affiliations:** 1 Department of Diabetes & Metabolic Diseases, Graduate School of Medicine, The University of Tokyo, Tokyo, Japan; 2 Data Science Office, Clinical Research Promotion Center, The University of Tokyo Hospital, Tokyo, Japan; 3 Department of Molecular and Medical Pharmacology, Faculty of Medicine, University of Toyama, Toyama, Japan; 4 Mitsubishi Corporation Life Sciences Limited, Tokyo, Japan; 5 Toranomon Hospital, Tokyo, Japan

**Keywords:** NAD^+^, Nicotinic acid, Nicotinamide, Age-related hearing loss

## Abstract

**Background:**

Age-related hearing loss (ARHL) is a common phenomenon observed during aging. On the other hand, the decrease in Nicotinamide adenine dinucleotide (NAD +) levels is reported to be closely related to the age-related declines in physiological functions such as ARHL in animal studies. Moreover, preclinical studies confirmed NAD + replenishment effectively prevents the onset of age-related diseases. However, there is a paucity of studies on the relationship between NAD^+^ metabolism and ARHL in humans.

**Methods:**

This study was analyzed the baseline results of our previous clinical trial, in which nicotinamide mononucleotide or placebo was administered to 42 older men (Igarashi et al., NPJ Aging 8:5, 2022). The correlations between blood levels of NAD^+^-related metabolites at baseline and pure-tone hearing thresholds at different frequencies (125, 250, 500, 1000, 2000, 4000, and 8000 Hz) in 42 healthy Japanese men aged > 65 years were analyzed using Spearman’s rank correlation. Multiple linear regression analysis was performed with hearing thresholds as the dependent variable and age and NAD^+^-related metabolite levels as independent variables.

**Results:**

Positive associations were observed between levels of nicotinic acid (NA, a NAD^+^ precursor in the Preiss-Handler pathway) and right- or left-ear hearing thresholds at frequencies of 1000 Hz (right: *r* = 0.480, *p* = 0.001; left: *r* = 0.422, *p* = 0.003), 2000 Hz (right: *r* = 0.507, *p* < 0.001, left: *r* = 0.629, *p* < 0.001), and 4000 Hz (left: *r* = 0.366, *p* = 0.029). Age-adjusted multiple linear regression analysis revealed that NA was an independent predictor of elevated hearing thresholds (1000 Hz (right): *p* = 0.050, regression coefficient (β) = 1610; 1000 Hz (left): *p* = 0.026, β = 2179; 2000 Hz (right): *p* = 0.022, β = 2317; 2000 Hz (left): *p* = 0.002, β = 3257). Weak associations of nicotinic acid riboside (NAR) and nicotinamide (NAM) with hearing ability were observed.

**Conclusions:**

We identified negative correlations between blood concentrations of NA and hearing ability at 1000 and 2000 Hz. NAD^+^ metabolic pathway might be associated with ARHL onset or progression. Further studies are warranted.

**Trial registration:**

The study was registered at UMIN-CTR (UMIN000036321) on 1st June 2019.

**Graphical Abstract:**

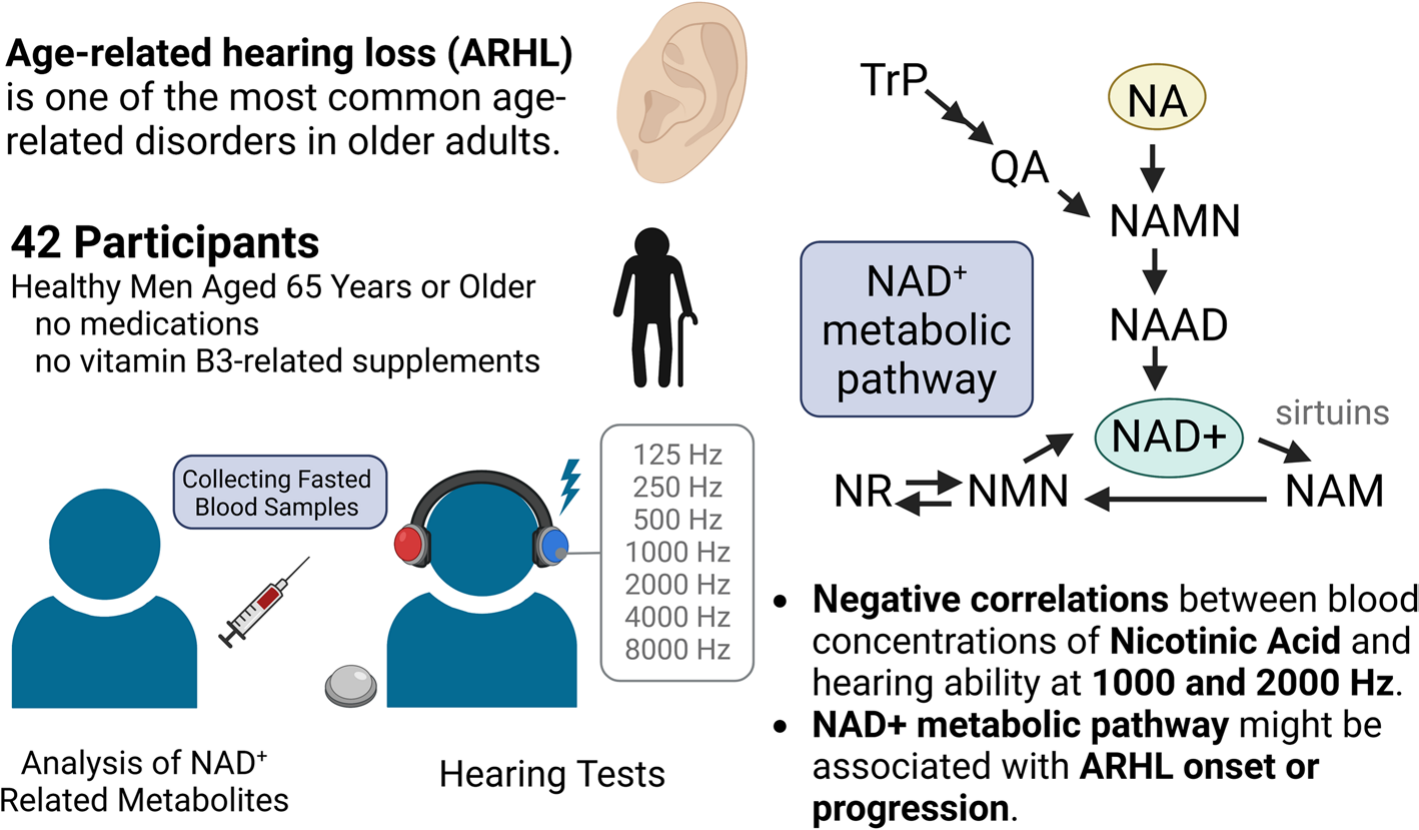

**Supplementary Information:**

The online version contains supplementary material available at 10.1186/s12877-023-03796-3.

## Introduction

Extension of healthy life expectancy has become a key challenge with the aging of the population. Age is a risk factor for several diseases, such as diabetes, cardiovascular disease, cancer, and Alzheimer's disease [1]. Aging and age-related diseases has been reported to be closely associated with age-related decline in blood and tissue nicotinamide adenine dinucleotide (NAD^+^) content [2–4]. Moreover, preclinical studies revealed that the administration of NAD + -related metabolites, such as nicotinamide (NAM), nicotinamide mononucleotide (NMN), or nicotinamide riboside (NR), reverses the aging-related decline in physiological function and prevents the onset of age-related diseases partly via the activation of NAD + dependent deacetylase sirtuin [2–4]. If the correlation between NAD + -related metabolites and any specific aging-related disorders in humans are demonstrated, the application of NAD + replenishment to them can be expected.

NAD + related-metabolites are derived from three main NAD^+^ biosynthetic pathways: the de novo pathway, Preiss-Handler pathway, and salvage pathway [5]. The Preiss-Handler pathway begins with nicotinic acid (NA), which originates from food or dietary supplements [5] or is produced by intestinal bacterial microflora [6]. NA is converted to nicotinic acid mononucleotide (NAMN) by nicotinate phosphoribosyltransferase (NAPRT) and nicotinic acid adenine dinucleotide (NAAD) to form NAD^+^ [5]. NAD^+^ catabolite NAM is converted to NMN in a rate-limiting step catalyzed by nicotinamide phosphoribosyltransferase (NAMPT) via the NAD^+^ salvage pathway [2–7].

Age-related hearing loss (ARHL) is one of the most common age-related disorders in older adults. ARHL is underscored by hearing impairments and speech communication disability caused by physiological age-related changes in the organum auditus and central nervous system circuits related to the auditory system. The World Health Organization defines disabling hearing loss as hearing loss greater than decibels hearing level (dB HL) in both ears, and hearing loss of 35 dB HL or greater in the worse ear [8]. The connection between NAD^+^ metabolism and ARHL has been identified in animals. SIRT3, one of the seven mammalian sirtuins, is a mitochondrial NAD^+^-dependent deacetylase that controls key metabolic pathways [9] and has been implicated in ARHL [10]. SIRT3 deacetylates mitochondrial isocitrate dehydrogenase 2 and increases the ratio of reduced-to-oxidized glutathione in mitochondria, thereby enhancing mitochondrial glutathione antioxidant defense system in mice [10]. Moreover, administration of NR, an intermediate NAD^+^ metabolite, activates the NAD^+^-SIRT3 pathway and prevents noise-induced hearing loss and spiral ganglia neurite degeneration in mice [11, 12]. Recently, we reported a placebo-controlled, randomized, double-blind, parallel-group trial in which 250 mg of NMN, another intermediate NAD^+^ metabolite, was administered to healthy older men daily for 6 or 12 weeks. Chronic NMN administration partially improved muscle performance (evaluated using gait speed and grip strength) in healthy older men and tended to improve audibility in the right ear, although the change was not statistically significant [13]. However, evidence for a correlation between ARHL and NAD^+^ metabolism in humans is limited.

Therefore, we aimed to investigate the correlation between NAD^+^ metabolites and hearing ability in older adults and analyzed the clinical data of older Japanese men that were obtained in our recent human clinical study [13].

## Materials and methods

### Study outline

This study was a sub analysis evaluating the association between NAD^+^-related metabolites and hearing ability using the baseline values obtained in our previous study [13]. The previous study was designed as a placebo-controlled, randomized, double-blind, parallel-group trial to evaluate the effects of NMN supplementation in older men [13].

The studies were approved by the Graduate School of Medicine and Faculty of Medicine, University of Tokyo Research Ethics Committee (2018013P) and registered as “Randomized, double-blind, placebo-controlled parallel-group comparative study to evaluate the effect of NMN on the body composition in elderly persons” at UMIN-CTR (UMIN000036321). 65 Japanese older men volunteers were recruited by NEWING NPO Corp (Tokyo, Japan), which is a third-party nonprofit organization. After some screening tests to assess eligibility for the NMN-supplementation study, in total 42 healthy Japanese men aged > 65 years were enrolled in this study. The details of the inclusion and exclusion criteria have been described in the previous study [13]. Candidates taking medications and vitamin B3-related supplements were excluded. The blood concentrations of NAD^+^ metabolites and aging-related parameters, including hearing ability, were measured and evaluated as secondary objectives.

All participants provided written informed consent to participate in the study. The study was conducted in accordance with the guidelines of the Declaration of Helsinki at the Clinical Research Support Center Phase 1 Unit of the University of Tokyo Hospital.

### Extraction of NAD^+^-related metabolites and LC–MS analysis 

Participants fasted and visited the Clinical Research Support Center Phase 1 Unit of the University of Tokyo Hospital in the morning. The blood samples were collected from forearms of the participants. The samples in heparinized tubes were frozen at -80 °C and analyzed at the University of Toyama. The extraction and analysis of NAD + related metabolites (NAD + , NA, nicotinic acid riboside (NAR), NANM, NMN, NR, and NAM) were performed as previously described [14].

50 µL of whole blood and 450 µL of 50% MeOH were mixed and vortexed for 10 s, followed by the addition of an equal volume of chloroform (500 μL). The mixture was centrifuged at 13,000 × g at 4 °C for 10 min, and then aqueous phase and organic phase were separated. The upper aqueous phase was transferred to a new tube, and this liquid–liquid extraction was repeated. After this procedure, the samples were dried. The dried aqueous phase was reconstituted in LC/MS-grade water. The metabolites were analyzed by using an Agilent 6460 Triple Quad mass spectrometer (Agilent Technologies Inc., Santa Clara, CA, USA) and Agilent 1290 HPLC system (Agilent Technologies Inc.). Analytes were separated on an Atlantis T3 column (2.1 × 150 mm, particle size 3 µm, Waters) using mobile phase A (5 mM ammonium formate) and mobile phase B (methanol) at a flow rate of 150 µL/min and column temperature of 40 °C. In addition, the programmed mobile phase gradient was following: 0–10 min, 0–70% B; 10–15 min, 70% B; 15–20 min, 0% B. Analysis of chromatograms were performed by using MassHunter Quantitative Analysis software (Agilent Technologies Inc.). Various concentrations of the standard compounds were measured to make standard curves for quantification.

### Hearing tests

Hearing ability of the right and left ears was measured by using an audiometer (Audiometer AA-79, RION Co., Ltd., Tokyo, Japan). Participants wore dedicated headphones in a quiet room and listened to acoustic pure-tone signals. If participants responded at 40 dB, the volume was lowered by 10–20 dB until no response was seen, and then the volume was gradually raised by 5 dB until participants responded. The volume level at which participants responded again was recognized as their hearing threshold. Besides, if there was no response at 40 dB, the volume was increased by 10–20 dB until a response was observed. Then, the volume was decreased by 10–20 dB until no response was seen, after that the volume was gradually increased by 5 dB until participants responded again. The volume level at which participants responded again was recognized as their hearing threshold. The same procedures were followed in order to measure and evaluate pure-tone air-conduction hearing thresholds at frequencies of 2000 Hz, 4000 Hz, 8000 Hz, 500 Hz, 250 Hz, and 125 Hz, in the order [15].

### Statistical analysis

**Fig. 1:**
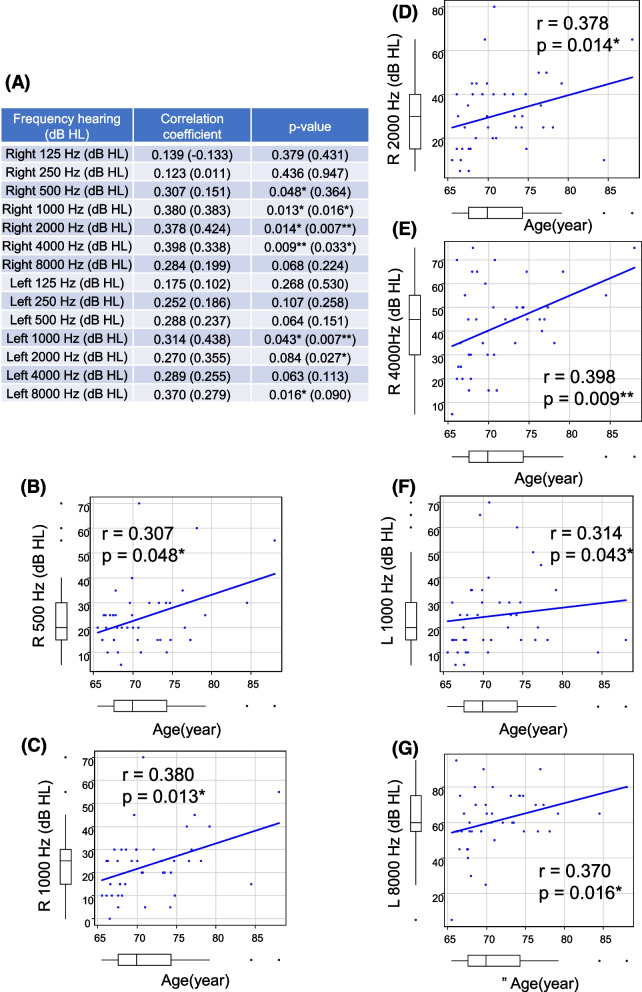
Correlation between age and hearing thresholds at each frequency. **A** Correlation of age (years) with right- and left-ear hearing thresholds at each frequency (dB HL). **B**-**E** Correlation between age and right-ear hearing thresholds at 500 Hz (**B**), 1000 Hz (**C**), 2000 Hz (**D**), and 4000 Hz (**E**). **F** and **G** Correlation between age and left-ear hearing thresholds at 1000 Hz (**F**) and 8000 Hz (**G**). Comparisons were performed using Spearman’s rank correlation coefficient. The correlation coefficients and *p*-values analyzed excluding outliers are presented in parentheses. *P < 0.05; **P < 0.01

**Fig. 2:**
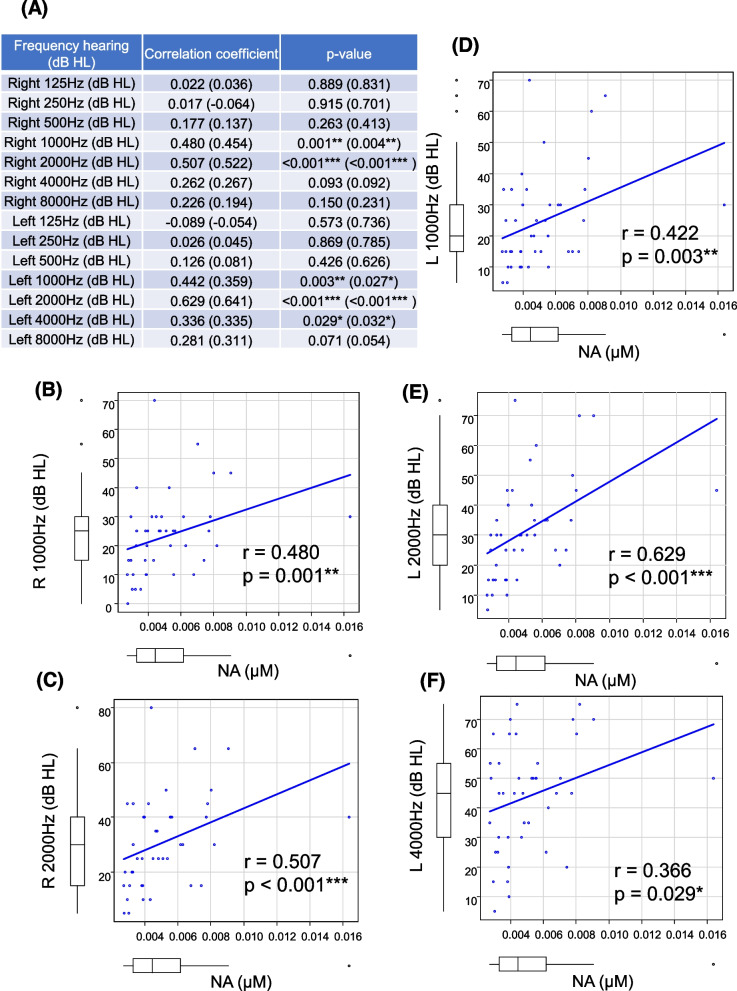
Correlation between NA (nicotinic acid) levels and hearing thresholds at each frequency. **A** Correlation of NA levels (μM) with right and left-ear hearing thresholds at each frequency (dB HL). **B** and **C** Correlation between NA levels and right-ear hearing thresholds at 1000 Hz (**B**) and 2000 Hz (**C**). **D**-**F** Correlation between NA levels and left-ear hearing thresholds at 1000 Hz (**D**), 2000 Hz (**E**), and 4000 Hz (**F**). Comparisons were performed using Spearman’s rank correlation coefficient. The correlation coefficients and *p*-values analyzed excluding outliers are presented in parentheses. *P < 0.05; **P < 0.01; ***P < 0.001

**Fig. 3:**
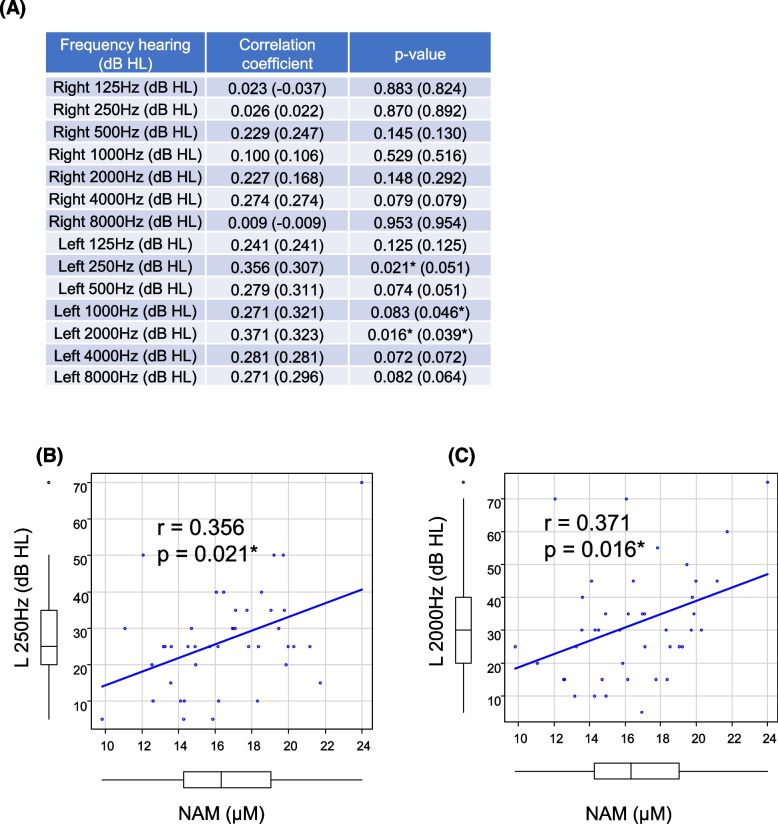
Correlation between the NAM (nicotinamide) levels and hearing thresholds at each frequency. **A** Correlation of NAM levels (μM) with right- or left-ear hearing thresholds at each frequency (dB HL). **B** and **C** Correlation between NAM levels and left-ear hearing thresholds at 250 Hz (**B**) and 2000 Hz (**C**). Comparisons were performed using Spearman’s rank correlation coefficient. The correlation coefficients and *p*-values analyzed excluding outliers are presented in parentheses. *P < 0.05

Statistical analysis of the data from the 42 participants was performed using Easy R for Microsoft Windows [16]. Correlations between age (year) or whole-blood concentrations of NAD^+^-related metabolites (µM) and hearing thresholds (dB HL) were analyzed using Spearman’s rank correlation coefficient. Multiple linear regression analysis was also performed to assess the age-adjusted correlation between hearing threshold (dB HL) and whole-blood concentrations of NAD^+^-related metabolites (µM) by designating hearing threshold as the dependent variable and NAD^+^-related metabolites and age as the independent variables. The regression coefficient represents the amount of hearing threshold (dB HL) when the NA (μM) changes by 1 μM. Outliers were defined as values outside of the third quartile + 1.5 × quartile range as the maximum and the first quartile -1.5 × quartile range as the minimum. The correlation coefficients and *p*-values analyzed by excluding outliers are presented in parentheses in Figs. 1, 2 and 3, Table 2, Supplementary Tables 1–5, and Supplementary Fig. 1. Statistical significance was set at *p* < 0.05.

## Results

### Participant characteristics 

Japanese men aged 65–88 years participated in the study. The clinical characteristics of the 42 participants, including the mean and SD of age, blood concentrations of NAD^+^-related metabolites (NAD^+^, NA, NAR, NANM, NMN, NR, and NAM), and hearing thresholds at each frequency, are presented in Table 1. An increase in mean thresholds at higher frequencies (2000, 4000, and 8000 Hz) was observed in both ears, consistent with previous data from older Japanese men [17].

**Table 1:** Clinical characteristics of 42 participants

	**Mean ± SD**
Age (year)	71.5 ± 5.1
NAD + (μM)	0.199 ± 0.073
NA (μM)	0.00512 ± 0.00248
NAR (μM)	0.000872 ± 0.000545
NANM (μM)	0.0715 ± 0.0972
NMN (μM)	0.0755 ± 0.0149
NR (μM)	0.0336 ± 0.0118
NAM (μM)	16.5 ± 3.1
Right 125 Hz (dB HL)	27.4 ± 11.9
Right 250 Hz (dB HL)	27.5 ± 13.8
Right 500 Hz (dB HL)	24.3 ± 13.2
Right 1000 Hz (dB HL)	23.3 ± 14.0
Right 2000 Hz (dB HL)	31.0 ± 16.8
Right 4000 Hz (dB HL)	42.5 ± 18.0
Right 8000 Hz (dB HL)	60.7 ± 16.7
Left 125 Hz (dB HL)	27.1 ± 11.5
Left 250 Hz (dB HL)	26.5 ± 13.6
Left 500 Hz (dB HL)	23.3 ± 13.2
Left 1000 Hz (dB HL)	24.8 ± 15.6
Left 2000 Hz (dB HL)	31.9 ± 16.9
Left 4000 Hz (dB HL)	44.0 ± 18.4
Left 8000 Hz (dB HL)	61.3 ± 17.4

The mean and standard deviation of age, NAD + related metabolites (NAD + , NA, NAR, NAMN, NMN, NR and NAM) and right or left-ear hearing thresholds at each frequency from the 42 participants

*NAD* + Nicotinamide adenine dinucleotide, *NA* Nicotinic acid, *NAR* Nicotinic acid riboside, *NAMN* Nicotinic acid mononucleotide, *NMN* Nicotinamide mononucleotide, *NR* Nicotinamide riboside, *NAM* Nicotinamide

### Relationship of age with hearing thresholds and whole-blood concentrations of NAD^+^-related metabolites

The relationship between age and hearing threshold at each frequency was assessed (Fig. 1A). Hearing thresholds have been reported to be progressively higher in older age at all frequencies [17]. Consistent with this, Spearman’s rank correlation tests revealed positive associations between age and right-ear hearing thresholds at 500 Hz (*r* = 0.307, *p* = 0.048) (Fig. 1B), 1000 Hz (*r* = 0.380, *p* = 0.013) (Fig. 1C), 2000 Hz (*r* = 0.378, *p* = 0.014) (Fig. 1D), and 4000 Hz (*r* = 0.398, *p* = 0.009) (Fig. 1E). Moreover, we observed a positive association between age and left-ear hearing thresholds at 1000 Hz (*r* = 0.314, *p* = 0.043) (Fig. 1F) and 8000 Hz (*r* = 0.370, *p* = 0.016) (Fig. 1G). However, no correlation was observed between age and blood concentrations of NAD^+^-related metabolites (Supplemental Table 1).

### Relationship of NA and NAR, precursors of the Preiss-Handler pathway, with hearing thresholds

Spearman’s rank correlation coefficient test was basically conducted to identify the correlation between NAD^+^ related metabolite levels and hearing thresholds at each frequency. Our data indicated that age and blood NAD^+^ levels were not correlated in participants aged more than 65 years. Nevertheless, an age-related decrease in NAD^+^ and reciprocal increase in plasma NAM has been reported among a sample of participants with a wide age range (20–87 years) [18]. Since age is a potential confounder, we also performed multiple linear regression analysis with hearing thresholds as the dependent variable and age and NAD^+^-related metabolite levels as the independent variables in the following analyses.

We first focused on the Preiss-Handler pathway, which uses dietary NA and NAPRT to generate NAMN that is subsequently transformed into NAAD by nicotinamide mononucleotide transferase (NMNAT) [5, 7]. The correlation between whole-blood concentrations of NA, the precursor of the Preiss–Handler pathway, and hearing thresholds was tested at each frequency (Fig. 2A). Spearman’s rank correlation tests revealed positive associations between NA levels and right-ear hearing thresholds at 1000 Hz (*r* = 0.480, *p* = 0.001) (Fig. 2B) and 2000 Hz (*r* = 0.507, *p* < 0.001) (Fig. 2C). Similarly, age-adjusted multiple linear regression analysis (dependent variable: right-ear hearing threshold, independent variable: NA and age) revealed that NA was an independent predictor of elevated right-ear hearing thresholds (1000 Hz: *p* = 0.050, regression coefficient (β) = 1610, 95% confidence intervals (CI) = 0.840 to 3220; 2000 Hz: *p* = 0.022, β = 2317, 95% CI = 348 to 4286) (Table 2). Moreover, Spearman’s rank correlation tests revealed positive associations between NA levels and left-ear hearing threshold at 1000 Hz (*r* = 0.422, *p* = 0.003) (Fig. 2D), 2000 Hz (*r* = 0.629, *p* < 0.001) (Fig. 2E), and 4000 Hz (*r* = 0.366, *p* = 0.029) (Fig. 2F). Age-adjusted multiple linear regression analysis (dependent variable: left-ear hearing threshold, independent variable: NA and age) revealed that NA was an independent predictor of elevated hearing thresholds in left ear (1000 Hz: *p* = 0.026, β = 2179, 95% CI = 271 to 4087; 2000 Hz: *p* = 0.002, β = 3257, 95% CI = 1306 to 5208) (Table 2).

**Table 2:** Multiple linear regression analysis with hearing threshold as the dependent variable and age and NAD + metabolites (NA, NAM, and NAR) as independent variables

Dependent variable	Independent variable	regression coefficient	95% lower confidence interval	95% upper confidence interval	*p*-value
(A) NA					
R 1000 Hz (dB HL)	NA (μM)	1610 (2657)	0.840 (934)	3220 (4379)	0.050* (0.004**)
R 2000 Hz (dB HL)	NA (μM)	2317 (3782)	348 (1585)	4286 (5979)	0.022* (0.001**)
L 1000 Hz (dB HL)	NA (μM)	2179 (2419)	271 (274)	4087 (4563)	0.026* (0.028*)
L 2000 Hz (dB HL)	NA (μM)	3257 (5968)	1306 (3777)	5208 (8159)	0.002** (< 0.001***)
L 4000 Hz (dB HL)	NA (μM)	1892 (3743)	-319 (519)	4103 (6966)	0.091 (0.024*)
(B) NAM					
L 250 Hz (dB HL)	NAM (μM)	2.00 (1.34)	0.752 (-0.00297)	3.26 (2.68)	0.002** (0.050)
L 2000 Hz (dB HL)	NAM (μM)	2.11 (1.43)	0.48 (-0.347)	3.75 (3.21)	0.013* (0.111)
(C) NAR					
R 125 Hz (dB HL)	NAR (μM)	-4504 (-14,605)	-10,882 (-31,840)	1875 (2631)	0.161 (0.094)
L 1000 Hz (dB HL)	NAR (μM)	6197 (7358)	-2783 (-14,109)	15,176 (28,826)	0.171 (0.489)
L 2000 Hz (dB HL)	NAR (μM)	10,156 (9963)	679 (-19,773)	19,633 (39,698)	0.036* (0.499)

(A, B and C) Multiple linear regression analysis with hearing threshold (dB HL) at each frequency as dependent variable and NA (μM) (A), NAM (μM) (B) or NAR (μM) (C) as independent variables, adjusted for age

The values analyzed excluding outliers are presented in parentheses. *P < 0.05; **P < 0.01; ***P < 0.001. R: Right, L: Left

In addition, the same analyses were performed on the data set excluding outliers. Outliers were defined as values outside of the third quartile + 1.5 × quartile range as the maximum and the first quartile -1.5 × quartile range as the minimum. Even after excluding outliers, Spearman’s rank correlation tests still presented positive associations between NA levels and right or left-ear hearing thresholds at 1000 Hz (right: *r* = 0.454, *p* = 0.004, left: 1000 Hz (*r* = 0.359, *p* = 0.027)),2000 Hz (right: *r* = 0.522, *p* < 0.001, left: *r* = 0.641, *p* < 0.001) and 4000 Hz (*r* = 0.335, *p* = 0.032) (Fig. 2A). Age-adjusted multiple linear regression analysis (dependent variable: right-ear hearing threshold, independent variable: NA and age) also revealed that NA was a still independent predictor of elevated right-ear hearing thresholds (1000 Hz (right): *p* = 0.004, β = 2657, 95% CI = 934 to 4379; 1000 Hz (left): *p* = 0.028, β = 2419, 95% CI = 274 to 4563; 2000 Hz (right): *p* = 0.001, β = 3782, 95% CI = 1585 to 5979; 2000 Hz (left): *p* < 0.001, β = 5968, 95% CI = 3777 to 8159; 4000 Hz (left): *p* = 0.024, β = 3743, 95% CI = 519 to 6966) (Table 2).

Further, we observed weak positive correlations of NA levels with right-ear hearing threshold at 4000 Hz (*r* = 0262, *p* = 0.093) and left-ear hearing threshold at 8000 Hz (*r* = 0281, *p* = 0.071) in Spearman’s rank correlation tests (Fig. 2A). However, we did not observe any positive correlation between NA levels and hearing thresholds at lower frequencies (Fig. 2A). Collectively, these findings indicated that NA levels were significantly correlated with hearing thresholds at 1000 Hz and 2000 Hz and that these correlations were not driven by outliers.

Next, we tested the correlation between the levels of NAR, which is converted from NA or NAMN in the Preiss-Handler pathway [5, 7], and hearing thresholds at each frequency (Supplemental Fig. 1A). Spearman’s rank correlation tests revealed associations of NAR with right- and left-ear hearing thresholds at 125 Hz (right: *r* = -0.396, *p* = 0.009) (Supplemental Fig. 1B), 1000 Hz (left: *r* = 0.335, *p* = 0.030) (Supplemental Fig. 1C), and 2000 Hz (left: *r* = 0.390, *p* = 0.011) (Supplemental Fig. 1D). Furthermore, age-adjusted multiple linear regression analysis (dependent variable: right- or left-ear hearing threshold, independent variable: NAR and age) revealed that NAR partly influence hearing thresholds (125 Hz (right): *p* = 0.161, β = -4504, 95% CI = -10,882 to 1875; 1000 Hz (left): *p* = 0.171, β = 6197, 95% CI = -2783 to 15,176; 2000 Hz (left): *p* = 0.036, β = 10,156, 95% CI = 679 to 19,633) (Table 2). No significant correlations were noted between NAR levels and hearing thresholds at other frequencies (Supplemental Fig. 1A). These data indicated that NAR levels were weakly correlated with hearing thresholds in older adults.

### Relationship of NAM, precursor of the salvage pathway, with hearing thresholds

We next focused on the salvage pathway that recycles NAM to NMN, which is further converted to NAD^+^ [2–7]. The relationship of whole-blood concentrations of NAM, precursor of the salvage pathway, with hearing thresholds at each frequency was tested (Fig. 3A). Spearman’s rank correlation tests revealed positive associations between NAM levels and left-ear hearing thresholds at 250 Hz (*r* = 0.356, *p* = 0.021) (Fig. 3B) and 2000 Hz (*r* = 0.371, *p* = 0.016) (Fig. 3C). Similarly, age-adjusted multiple linear regression analysis (dependent variable: left-ear hearing threshold, independent variable: NAM and age) revealed that NAM was an independent predictor of elevated left-ear hearing thresholds (250 Hz: *p* = 0.002, β = 2.00, 95% CI = 0.752 to 3.26; 2000 Hz: *p* = 0.013, β = 2.11, 95% CI = 0.48 to 3.75) (Table 2). Spearman’s rank correlation tests revealed weak positive correlations of NAM with right-ear hearing threshold at 4000 Hz (*r* = 0.274, *p* = 0.079) and left-ear hearing thresholds at 500 Hz (*r* = 0.279, *p* = 0.074), 1000 Hz (*r* = 0.271, *p* = 0.083), 4000 Hz (*r* = 0.281, *p* = 0.072), and 8000 Hz (*r* = 0.271, *p* = 0.082) (Fig. 3A). These data indicated that NAM levels correlated weakly with hearing threshold in older adults.

### Other NAD^+^-related metabolites and hearing thresholds

Finally, we analyzed the correlations of whole-blood concentrations of NAD^+^ and other NAD^+^-related metabolites (NAMN, NMN, and NR) with hearing thresholds at each frequency using Spearman’s rank correlation tests (Supplemental Tables 2–5). Spearman’s rank correlation tests revealed no significant correlations between NAD^+^ and hearing thresholds at each frequency (Supplemental Table 2) or between NAMN, NMN, NR, and hearing thresholds at each frequency (Supplemental Tables 3–5). These data indicated that NAD^+^-related metabolites other than NA, NAR, and NAM were not correlated with hearing ability in older adults.

## Discussion

The relationship between NAD^+^-related metabolite levels and hearing ability in older adults has not been reported to date. In this study, we report that whole-blood concentrations of NA and NAD^+^ precursors correlate positively with hearing thresholds, which indicates the metabolites correlate negatively with hearing ability in older men. Our analyses cannot determine if the link between NA levels and healing ability is causation or association. Nevertheless, we hypothesize as described below about what the correlation means. Moreover, we suppose that blood concentrations of NA could be a marker of hearing ability in older adults.

In this study, we observed a negative correlation between concentrations of NA, an NAD^+^ precursor in the Preiss-Handler pathway, and hearing ability at 1000, 2000, and 4000 Hz. In contrast, concentrations of NAMN, which is generated from NA by NAPRT, did not correlate with hearing thresholds. Based on these results, we conjecture that the enzymatic activity of NAPRT, which converts NA into NAMN [5, 7], may determine the amount of NAD^+^ in the inner ear. Moreover, since NAM, an NAD^+^ precursor in the salvage pathway [2–7], was associated with hearing ability, we propose that the contribution of NAMPT to the maintenance of NAD^+^ levels in the inner ear may be limited compared to that of NAPRT. The enzyme NAPRT was first identified in human erythrocytes by Handler, whereby it increased NAD^+^ levels [19]. Although enzymatic activity has been detected in most mouse tissues [20], NAPRT activity can be tissue-specific. Although NAM levels are substantially higher than NA levels throughout mammalian living cells, some tissues preferentially use NA for NAD^+^ synthesis [21]. NA acts as a more efficient precursor than NAM in the mouse liver, intestine, heart, and kidneys [22, 23]. Based on the stronger correlation between the level of NA and hearing ability compared with that of NAM, we speculate that the Preiss-Handler pathway may constitute a major source of NAD^+^ in the inner ear relative to the Salvage pathway.

Interestingly, the relationships between NA levels and hearing thresholds were most evident at 1000 and 2000 Hz, which tend to be less elevated with age than the higher frequencies [17]. Hearing loss at low frequency can be caused by damage to the hair cells, cochlear, or auditory nerve [24]. With the protective role of cochlear hair cells by the mitochondrial surtuin SIRT3 [10, 11], we speculate NAD + -SIRT3 pathway might be the main pathway by which NAD + metabolism is involved with hearing ability in humans. Administration of NR activates the NAD^+^-SIRT3 pathway and prevents noise-induced hearing loss and spiral ganglion process degeneration in mice [11, 12]. Based on preclinical findings [10–12], activation of NAD^+^ metabolism could similarly affect hearing in humans through mechanisms including activation of SIRT3 and increased reduced-to-oxidized glutathione ratio in mitochondria. Actually, we previously reported that chronic administration of NMN 250 mg/day had a tendency to improve audibility in healthy older men [13]. Our findings in this report suggest that NAD^+^ metabolic pathway, in particular the Preiss-Handler pathway might be associated with the development of ARHL in humans. If the loss of the enzyme activity of NAPRT, the dominant enzyme in Preiss-Handler pathway, is the determinant of ARHL, NAD^+^ replenishment through the different pathway from Preiss-Handler pathway such as NMN administration can effectively contribute to ARHL improvement.

We did not observe any correlation between whole-blood levels of NAD^+^ and hearing ability in our study. This could be due to the instability of NAD^+^ in the blood, making it technically difficult to detect whole-blood NAD^+^ [25]. In this regard, we may have identified a correlation between tissue NAD^+^ levels and hearing ability by measuring NAD^+^ levels in the inner ear. As NAM levels increase with the breakdown of NAD^+^ in the salvage pathway [6, 18], the negative correlation between NAM and hearing ability implies a positive correlation between NAD^+^ and hearing ability.

This study has several limitations. This study was sub-analysis, and the sample size was not pre-calculated for this correlation analysis. Although some correlations might be undetectable due to the small sample size in our analysis, post-hoc power calculation for spearman’s rank correlation showed that the sample size was enough to detect the moderate or strong correlations (power = 0.900 (correlation coefficient = 0.5), and power = 0.702 (correlation coefficient = 0.4)). Additionally, participants comprised healthy Japanese men aged 65 years or older. The results may be relevant only for the certain healthy older Japanese men. Further investigations including other races, generation, and gender are warranted. Moreover, as all analyses were exploratory, multiple comparisons were not performed. However, the significant correlation between NA levels and hearing ability were observed at the same frequencies (1000 and 2000 Hz) on both ear sides in our analyses. While this consistency is not explained by false positive results, we consider our findings deserves further investigation to confirm our findings. Finally, the mechanism of ARHL requires further elucidation from the perspective of the NAD^+^ metabolic pathway, including the Preiss-Handler pathway and NAD^+^-SIRT3 axis [10–12] in basic and clinical research.

In conclusion, we report a negative correlation between whole-blood concentrations of NA and hearing ability. The relevance of NAD^+^ metabolic pathways, particularly the Preiss-Handler pathway, to the development of ARHL should be further investigated in future studies.

## Supplementary Information

**Additional file 1: Supplementary Table 1.** The correlations between age and the level of NAD+ related metabolites. The correlation between age (year) and NAD+ related metabolites (μM). Comparisons were made using the Spearman’s rank correlation coefficient. The correlation coefficients and *p*-values analyzed excluding outliers are presented in parentheses. **Supplementary Table 2.** The correlation between the level of NAD+ and hearing thresholds at each frequency. The correlation between the level of NAD+ (μM) and right or left-ear hearing thresholds at each frequency (dB HL). Comparisons were made using the Spearman’s rank correlation coefficient. The correlation coefficients and *p*-values analyzed excluding outliers are presented in parentheses. **Supplementary Table 3.** The correlation between the level of NAMN and hearing thresholds at each frequency. The correlation between the level of NAMN (μM) and right or left-ear hearing thresholds at each frequency (dB HL). Comparisons were made using the Spearman’s rank correlation coefficient. The correlation coefficients and *p*-values analyzed excluding outliers are presented in parentheses. **Supplementary Table 4.** The correlation between the level of NMN and hearing thresholds at each frequency. The correlation between the level of NMN (μM) and right or left-ear hearing thresholds at each frequency (dB HL). Comparisons were made using the Spearman’s rank correlation coefficient. The correlation coefficients and *p*-values analyzed excluding outliers ae presented in parentheses. **Supplementary Table 5.** The correlation between the level of NR and hearing thresholds at each frequency. The correlation between the level of NR (μM) and right or left-ear hearing thresholds at each frequency (dB HL). Comparisons were made using the Spearman’s rank correlation coefficient. The correlation coefficients and *p*-values analyzed excluding outliers are presented in parentheses. **Supplemental Figure 1.** The correlation between the level of NAR and hearing thresholds at each frequency. (A) The correlation between the level of NAR (μM) and right or left-ear hearing thresholds at each frequency (dB HL). (B) The correlation between the level of NAR and right-ear hearing thresholds at 250Hz. (C and D) The correlation between the level of NAR and left-ear hearing thresholds at 1000Hz(C) and 2000Hz(D). Comparisons were made using the Spearman’s rank correlation coefficient. The correlation coefficients and *p*-values analyzed excluding outliers are presented in parentheses. *P < 0.05; **P < 0.01

## Data Availability

The datasets generated and/or analyzed during this study are available from the corresponding author upon reasonable request.

## Abbreviations

95% CI: 95% Confidence intervals
ARHL: Age-related hearing loss
NAAD: Nicotinic acid adenine dinucleotide
NA: Nicotinic acid
NAD^+^: Nicotinamide adenine dinucleotide
NAM: Nicotinamide
NAMN: Nicotinic acid mononucleotide
NAMPT: Nicotinamide phosphoribosyltransferase
NAPRT: Nicotinate phosphoribosyltransferase
NAR: Nicotinic acid riboside
NMN: Nicotinamide mononucleotide
NR: Nicotinamide riboside
