# Supplementary material for: Blood levels of nicotinic acid negatively correlate with hearing ability in healthy older men
Source: BMC Geriatr. 2023 Feb 15;23:97. doi: 10.1186/s12877-023-03796-3 (PMC9933288; doi:10.1186/s12877-023-03796-3)
Supplement: Supplementary file 1 — Additional file 1: Supplementary Table 1. The correlations between age and the level of NAD+ related metabolites. The correlation between age (year) and NAD+ related metabolites (μM). Comparisons were made using the Spearman’s rank correlation coefficient. The correlation coefficients and p-values analyzed excluding outliers are presented in parentheses. Supplementary Table 2. The correlation between the level of NAD+ and hearing thresholds at each frequency. The correlation between the level of NAD+ (μM) and right or left-ear hearing thresholds at each frequency (dB HL). Comparisons were made using the Spearman’s rank correlation coefficient. The correlation coefficients and p-values analyzed excluding outliers are presented in parentheses. Supplementary Table 3. The correlation between the level of NAMN and hearing thresholds at each frequency. The correlation between the level of NAMN (μM) and right or left-ear hearing thresholds at each frequency (dB HL). Comparisons were made using the Spearman’s rank correlation coefficient. The correlation coefficients and p-values analyzed excluding outliers are presented in parentheses. Supplementary Table 4. The correlation between the level of NMN and hearing thresholds at each frequency. The correlation between the level of NMN (μM) and right or left-ear hearing thresholds at each frequency (dB HL). Comparisons were made using the Spearman’s rank correlation coefficient. The correlation coefficients and p-values analyzed excluding outliers ae presented in parentheses. Supplementary Table 5. The correlation between the level of NR and hearing thresholds at each frequency. The correlation between the level of NR (μM) and right or left-ear hearing thresholds at each frequency (dB HL). Comparisons were made using the Spearman’s rank correlation coefficient. The correlation coefficients and p-values analyzed excluding outliers are presented in parentheses. Supplemental Figure 1. The correlation between the level of NAR and he [file 12877_2023_3796_MOESM1_ESM.pdf]

Supplementary Table 1.

The correlations between age and the level of NAD+ related metabolites.

NAD+-related metabolites(μM)	Correlation coefficient	p-value
NAD+	0.163 (0.168)	0.303 (0.301)
NAM	-0.029 (0.008)	0.857 (0.961)
NA	0.211 (0.166)	0.181 (0.312)
NAMN	-0.004 (-0.025)	0.980 (0.888)
NAR	-0.004 (-0.086)	0.982 (0.630)
NMN	-0.058 (-0.080)	0.714 (0.628)
NR	0.200 (0.130)	0.204 (0.448)

The correlation between age (year) and NAD+ related metabolites (μM). Comparisons were made using the Spearman's rank correlation coefficient. The correlation coefficients and p-values analyzed excluding outliers are presented in parentheses.

Supplementary Table 2.

The correlation between the level of NAD+ and hearing thresholds at each frequency.

Frequency hearing (dB HL)	Correlation coefficient	p-value
Right 125Hz (dB HL)	0.192 (0.107)	0.223 (0.523)
Right 250Hz (dB HL)	0.173 (0.191)	0.273 (0.238)
Right 500Hz (dB HL)	0.027 (-0.038)	0.868 (0.819)
Right 1000Hz (dB HL)	-0.011 (-0.018)	0.945 (0.915)
Right 2000Hz (dB HL)	0.104 (0.115)	0.511 (0.478)
Right 4000Hz (dB HL)	-0.011 (-0.087)	0.943 (0.588)
Right 8000Hz (dB HL)	0.136 (0.122)	0.391 (0.454)
Left 125Hz (dB HL)	0.012 (-0.029)	0.942 (0.858)
Left 250Hz (dB HL)	-0.035 (0.002)	0.828 (0.989)
Left 500Hz (dB HL)	-0.049 (-0.111)	0.760 (0.503)
Left 1000Hz (dB HL)	-0.077 (-0.090)	0.627 (0.591)
Left 2000Hz (dB HL)	-0.117 (-0.023)	0.460 (0.889)
Left 4000Hz (dB HL)	-0.145 (-0.161)	0.359 (0.315)
Left 8000Hz (dB HL)	-0.077 (-0.191)	0.630 (0.243)

The correlation between the level of NAD+ (μM) and right or left-ear hearing thresholds at each frequency (dB HL).
Comparisons were made using the Spearman's rank correlation coefficient.
The correlation coefficients and p-values analyzed excluding outliers are presented in parentheses.

Supplementary Table 3.
The correlation between the level of NAMN and hearing thresholds at each frequency.

Frequency hearing (dB HL)	Correlation coefficient	p-value
Right 125Hz (dB HL)	-0.202 (-0.081)	0.201 (0.667)
Right 250Hz (dB HL)	-0.101 (0.021)	0.524 (0.910)
Right 500Hz (dB HL)	-0.100 (-0.141)	0.528 (0.441)
Right 1000Hz (dB HL)	0.229 (-0.037)	0.145 (0.838)
Right 2000Hz (dB HL)	0.170 (0.028)	0.281 (0.875)
Right 4000Hz (dB HL)	-0.082 (-0.225)	0.604 (0.193)
Right 8000Hz (dB HL)	0.073 (0.084)	0.646 (0.637)
Left 125Hz (dB HL)	-0.232 (-0.231)	0.140 (0.182)
Left 250Hz (dB HL)	-0.259 (-0.407)	0.097 (0.017*)
Left 500Hz (dB HL)	-0.254 (-0.380)	0.105 (0.027*)
Left 1000Hz (dB HL)	0.024 (-0.128)	0.879 (0.477)
Left 2000Hz (dB HL)	0.036 (-0.323)	0.820 (0.062)
Left 4000Hz (dB HL)	-0.079 (-0.200)	0.617 (0.249)
Left 8000Hz (dB HL)	-0.158 (-0.278)	0.319 (0.112)

The correlation between the level of NAMN (μM) and right or left-ear hearing thresholds at each frequency (dB HL).
Comparisons were made using the Spearman’s rank correlation coefficient.
The correlation coefficients and p-values analyzed excluding outliers are presented in parentheses.

Supplementary Table 4.

The correlation between the level of NMN and hearing thresholds at each frequency.

Frequency hearing (dB HL)	Correlation coefficient	p-value
Right 125Hz (dB HL)	-0.115 (-0.319)	0.470 (0.054)
Right 250Hz (dB HL)	0.036 (-0.051)	0.819 (0.758)
Right 500Hz (dB HL)	0.097 (-0.0004)	0.542 (0.998)
Right 1000Hz (dB HL)	0.008 (-0.057)	0.962 (0.730)
Right 2000Hz (dB HL)	0.155 (0.178)	0.327 (0.272)
Right 4000Hz (dB HL)	-0.060 (-0.052)	0.704 (0.748)
Right 8000Hz (dB HL)	0.122 (0.253)	0.443 (0.115)
Left 125Hz (dB HL)	0.029 (-0.012)	0.856 (0.941)
Left 250Hz (dB HL)	-0.069 (-0.077)	0.665 (0.637)
Left 500Hz (dB HL)	-0.158 (-0.195)	0.318 (0.234)
Left 1000Hz (dB HL)	-0.125 (-0.104)	0.432 (0.536)
Left 2000Hz (dB HL)	0.036 (0.053)	0.819 (0.745)
Left 4000Hz (dB HL)	-0.099 (-0.100)	0.533 (0.533)
Left 8000Hz (dB HL)	-0.086 (-0.098)	0.590 (0.555)

The correlation between the level of NMN (μM) and right or left-ear hearing thresholds at each frequency (dB HL).
Comparisons were made using the Spearman's rank correlation coefficient.
The correlation coefficients and p-values analyzed excluding outliers are presented in parentheses.

Supplementary Table 5.

The correlation between the level of NR and hearing thresholds at each frequency.

Frequency hearing (dB HL)	Correlation coefficient	p-value
Right 125Hz (dB HL)	-0.096 (-0.065)	0.545 (0.717)
Right 250Hz (dB HL)	-0.034 (-0.081)	0.831 (0.637)
Right 500Hz (dB HL)	-0.030 (-0.146)	0.849 (0.403)
Right 1000Hz (dB HL)	0.040 (-0.196)	0.802 (0.252)
Right 2000Hz (dB HL)	0.089 (-0.152)	0.577 (0.377)
Right 4000Hz (dB HL)	0.094 (-0.161)	0.553 (0.342)
Right 8000Hz (dB HL)	0.077 (-0.162)	0.627 (0.345)
Left 125Hz (dB HL)	0.256 (0.209)	0.102 (0.215)
Left 250Hz (dB HL)	0.285 (0.235)	0.067 (0.168)
Left 500Hz (dB HL)	0.151 (0.071)	0.341 (0.679)
Left 1000Hz (dB HL)	0.125 (-0.045)	0.430 (0.799)
Left 2000Hz (dB HL)	0.030 (-0.141)	0.852 (0.411)
Left 4000Hz (dB HL)	0.280 (0.093)	0.073 (0.583)
Left 8000Hz (dB HL)	0.230 (-0.028)	0.143 (0.875)

The correlation between the level of NR (μM) and right or left-ear hearing thresholds at each frequency (dB HL).
Comparisons were made using the Spearman’s rank correlation coefficient.
The correlation coefficients and p-values analyzed excluding outliers are presented in parentheses.

Supplemental Figure 1.

The correlation between the level of NAR and hearing thresholds at each frequency.

(A)

Frequency hearing (dB HL)	Correlation coefficient	p-value
Right 125Hz (dB HL)	-0.396 (-0.274)	0.009** (0.129)
Right 250Hz (dB HL)	-0.265 (-0.290)	0.089 (0.097)
Right 500Hz (dB HL)	-0.003 (-0.078)	0.983 (0.666)
Right 1000Hz (dB HL)	0.272 (0.106)	0.081 (0.553)
Right 2000Hz (dB HL)	0.250 (0.066)	0.110 (0.705)
Right 4000Hz (dB HL)	0.151 (-0.012)	0.339 (0.946)
Right 8000Hz (dB HL)	0.071 (-0.071)	0.655 (0.685)
Left 125Hz (dB HL)	-0.055 (-0.038)	0.732 (0.827)
Left 250Hz (dB HL)	-0.006 (-0.161)	0.969 (0.356)
Left 500Hz (dB HL)	-0.060 (-0.201)	0.708 (0.247)
Left 1000Hz (dB HL)	0.335 (0.164)	0.030* (0.355)
Left 2000Hz (dB HL)	0.390 (0.148)	0.011* (0.396)
Left 4000Hz (dB HL)	0.275 (0.149)	0.078 (0.387)
Left 8000Hz (dB HL)	0.131 (-0.012)	0.409 (0.947)

(C)

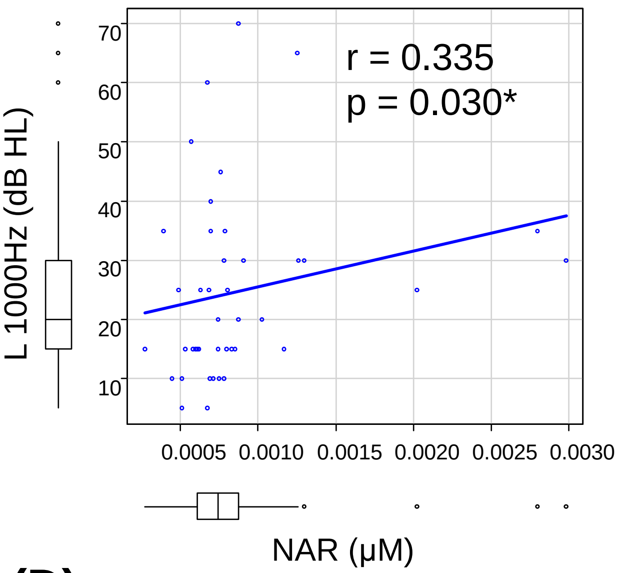

(B)

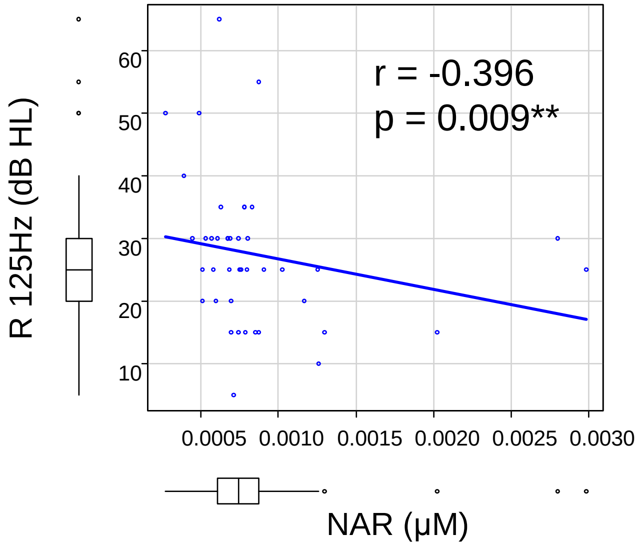

(D)

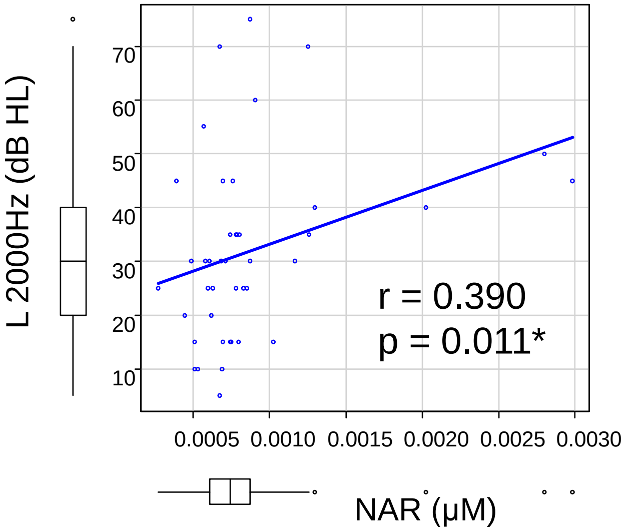

(A) The correlation between the level of NAR (μM) and right or left-ear hearing thresholds at each frequency (dB HL).
(B) The correlation between the level of NAR and right-ear hearing thresholds at 250Hz.
(C and D) The correlation between the level of NAR and left-ear hearing thresholds at 1000Hz(C) and 2000Hz(D).
Comparisons were made using the Spearman's rank correlation coefficient. The correlation coefficients and p-values analyzed excluding outliers are presented in parentheses.
*P < 0.05; **P < 0.01
